# Evidence for coloration plasticity in the yellow‐bellied toad, *Bombina variegata*

**DOI:** 10.1002/ece3.8391

**Published:** 2021-11-30

**Authors:** Kathleen Preißler, Ariel Rodríguez, Heike Pröhl

**Affiliations:** ^1^ Molecular Evolution and Systematics of Animals Institute of Biology University Leipzig Leipzig Germany; ^2^ Institute of Zoology University of Veterinary Medicine of Hannover Hannover Germany

**Keywords:** *Bombina variegata*, brightness, defensive coloration, phenotypic adaptation, spectral reflectance, visual modeling

## Abstract

Phenotypic adaptation in terms of background color matching to the local habitat is an important mechanism for survival in prey species. Thus, intraspecific variation in cryptic coloration is expected among localities with dissimilar habitat features (e.g., soil, vegetation). Yellow‐bellied toads (*Bombina variegata*) display a dark dorsal coloration that varies between populations, assumed to convey crypsis. In this study, we explored I) geographic variation in dorsal coloration and II) coloration plasticity in *B*. *variegata* from three localities differing in substrate coloration. Using avian visual modeling, we found that the brightness contrasts of the cryptic dorsa were significantly lower on the local substrates than substrates of other localities. In experiments, individuals from one population were able to quickly change the dorsal coloration to match a lighter substrate. We conclude that the environment mediates an adaptation in cryptic dorsal coloration. We suggest further studies to test the mechanisms by which the color change occurs and explore the adaptive potential of coloration plasticity on substrates of varying brightness in *B*. *variegata* and other species.

## Graphical abstract

The yellow‐bellied toad (*Bombina variegata*) displays a cryptic dorsal coloration pattern, which varies between localities. It is yet unknown whether this pattern is plastic or a response to local adaptation. Via visual modeling, we provide evidence for coloration plasticity in dorsal coloration in this amphibian species.

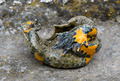

## 1 INTRODUCTION

The force of natural selection leads to geographic variation in traits that constitute advantages in ecologically dissimilar habitats in terms of fitness and survival. Geographic variation occurs in coloration‐based survival strategies like aposematism and crypsis that have evolved in prey species to evade predation (Mappes et al., 2005). These two strategies target either a reduced visibility of the prey by contrast reduction (crypsis, camouflage [Cuthill, 2019]—e.g., *Biston betularia* [Lees & Creed, 1975]) to the surrounding environment or an increased visibility by conspicuous, highly contrasting coloration (aposematism [Stevens & Ruxton, 2012]—for example, *Oophaga pumilio* [Dreher et al., 2015]). The latter is frequently associated with higher detection rates in predators, which are compensated by toxic components conveying unprofitability (Mappes et al., 2005).

Camouflage is likely the most widespread defense strategy in animals (Cuthill, 2019; Duarte et al., 2017). The contrast reduction in optimal camouflage can be achieved by various mechanisms. Short‐term color change for instance enables to swiftly match heterogenous habitat structures and decrease a prey's detectability as observed in some amphibian (Sanchez et al., 2019; Wente & Phillips, 2003), fish (Nilsson Sköld et al., 2013), and reptilian species (Teyssier et al., 2015). Many amphibians for instance get darker on black background owing to dispersal of melanin‐containing organelles (melanosomes) or aggregation of iridophores (cells with platelets involved in structural coloration), while they get lighter on light backgrounds. Long‐term adaptation in coloration to local environments that evolved in response to natural selection is reported from lizard (*Phyrnocephalus versicolor*, Tong et al., 2019) and mice populations (*Chaetodipus intermedius*; Hoekstra et al., 2005) that show geographically diverged body colorations to maximize camouflage on the local backgrounds. Furthermore, individuals can decrease detectability through behavioral strategies like substrate selection (Smithers et al., 2018).

The yellow‐bellied toad (*Bombina variegata*) displays a cryptic dorsal coloration varying in different shades of gray to brown and a conspicuous ventral yellow coloration with contrasting black patches (Kwet, 2015). The aposematic ventral display in *B*. *variegata* is a qualitative honest signal, indicating its unprofitability by the sequestration of skin poison (Kiss & Michl, 1962). The yellow‐bellied toad inhabits areas with lighter to darker substrates in terrestrial and aquatic habitats and varies in dorsal coloration among individuals and populations (Gollmann & Goldmann, 2012). The latter was also shown for *Bombina orientalis* (Kang et al., 2017). Therefore, it is an ideal study system to investigate whether frogs are adapted in dorsal coloration to their local habitat and/or whether they are able to change their coloration quickly when moved to another habitat with divergent substrate coloration. As *B*. *variegata* is endangered in Germany (Kühnel et al., 2009) and several populations suffer from small population sizes (Pröhl et al., 2021), these questions become relevant when individuals are transferred to depleted populations in order to increase genetic diversity. If these individuals show low plasticity in dorsal coloration, they might be subject to higher predation pressures, diminishing the success of individual reintroductions. The aim of this study was (1) to explore the extent of geographic variation in the dorsal coloration in yellow‐bellied toads and whether their coloration matches their local backgrounds to enhance crypsis, and (2) to test whether background matching is a plastic response to the local environment. Since the ventral yellow coloration is probably achieved through carotenoid pigments in xanthophores as in *B*. *orientalis* (Frost & Robinson, 1984) and carotenoids are ingested with the diet, we do not expect the ventral coloration to covary with the background.

## 2 MATERIALS AND METHODS

For this study, we combined measurements of body coloration of yellow‐bellied toads in the field with one experiment in the laboratory. The measurements in the field were used to test whether the dorsal and ventral coloration of frogs from three populations were adapted to the local substrate. In the laboratory experiment, we tested whether the frogs were able to change their coloration when transferred to another (lighter or darker) substrate. We explain the details in the following paragraphs:

### 2.1 Field measurements

**FIGURE 1:**
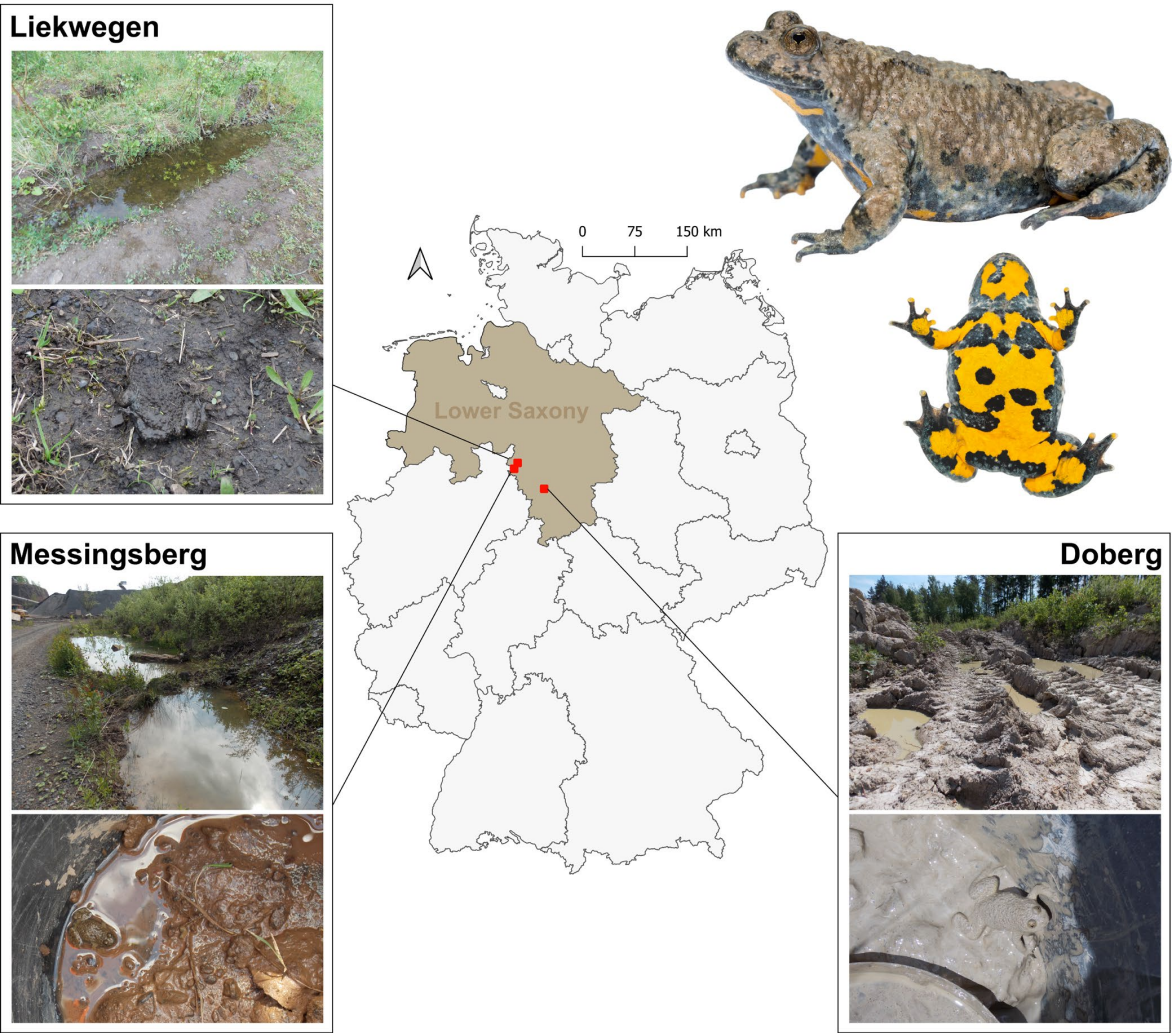
Localities (Liekwegen, Messingsberg, and Doberg) of the field measurements within yellow‐bellied toad populations in Germany. Pictures depicting exemplary habitat structures and frogs on substrate from each locality. Figure created using QGIS 3 and CorelDraw7. Pictures in upper right by courtesy of Konrad Kürbis, in upper left by Mirjam Nadjafzadeh

**FIGURE 2:**
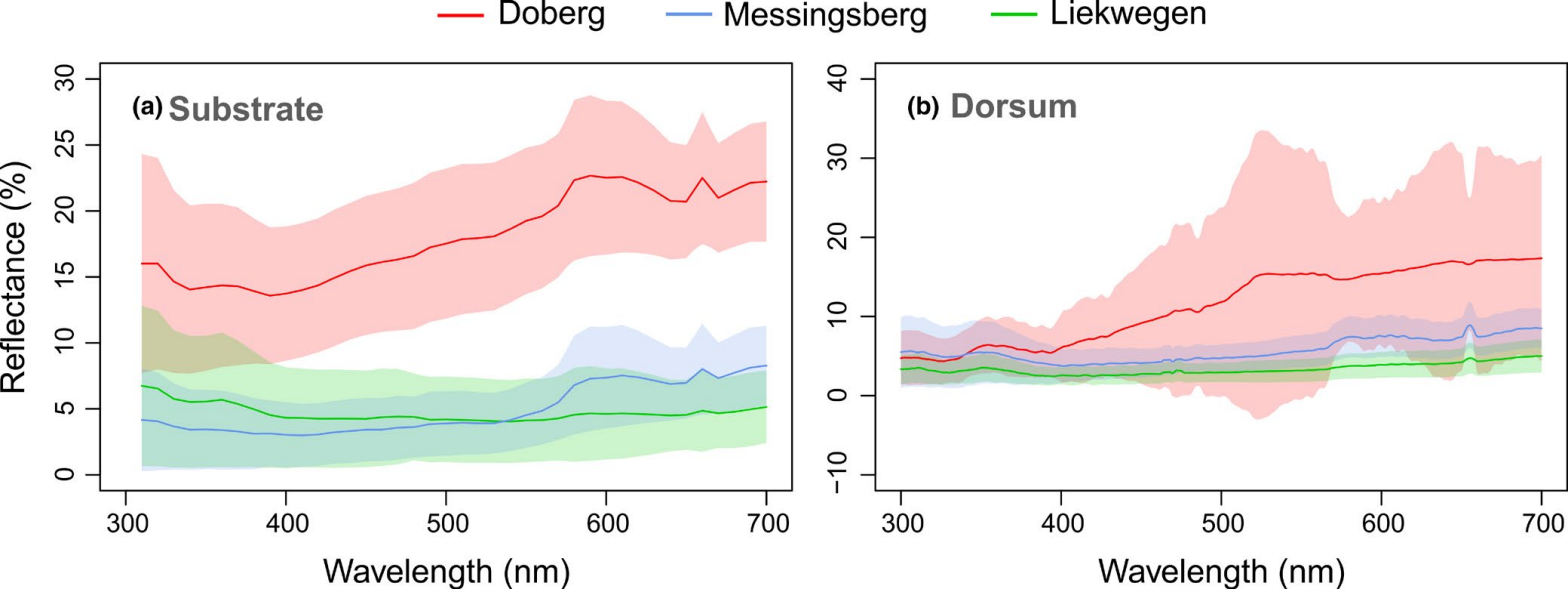
Spectral reflectance (%) for the visible range of wavelengths (nm) of the (a) pond substrates and the (b) dorsal side of *B*. *variegata* frogs from Doberg (red), Liekwegen (green), and Messingsberg (blue). Lines represent the averaged spectral reflectance for each wavelength and the shades mark the standard deviation. The graphs were created using the R package “*pavo*” (Maia et al., 2013)

In 2014, we studied three populations of *B*. *variegata* in southern Lower Saxony, Germany, that differed in the coloration of the natural substrate: Liekwegen (N 52°17′, E 09°11′, dark gray substrate), Messingsberg (N 52°13′, E 09°07′, reddish substrate), and Doberg (N 51°59′, E 09°41′, light gray substrate, Figure 1). All three study localities present secondary habitats in former (Liekwegen, Doberg) or still active (Messingsberg) quarries with sandy substrates. Since the distance between Liekwegen and Messingsberg is 8.1 km, Liekwegen and Doberg 46.7 km, and Doberg and Liekwegen 44.7 km, we assume that gene flow between localities is restricted (see Pröhl et al., 2021). We sampled a total number of 46 adults (Liekwegen *n* = 17, Messingsberg *n* = 14, and Doberg *n* = 16,) over two weeks in May and June by capturing the frogs from different ponds distributed across localities with a dip net. We measured the spectral reflectance of the frogs's dorsum as well as the substrates within and around the ponds in which the frogs were found (Figure 2). In each case, the substrates at the bottom of the pool were the same as the terrestrial substrates surrounding the ponds. We released the animals to the location of capture directly after measurements.

We used a bifurcated optical fiber (Ocean Optics R‐200‐7‐UV/VIS, fiber core size: 200 µm) connected to a spectrometer (Ocean Optics HR2000+) and a deuterium–tungsten DT‐Mini‐2GS lamp (Ocean Optics) to measure spectral reflectance. On every frog, we measured six different points of the grayish dorsum. All measuring points were evenly distributed over the dorsum. The spectroscopy software OceanView was applied while measuring and averaging five spectral scans with an integration time of 200 ms and a boxcar width of 5. Additionally, we measured the reflectance of six samples of pond substrates per captured frog. Before measuring each frog, we used the WS‐1‐SS white reflectance standard (diffuse reflectance standard, Ocean Optics) and a dark standard (light of DT‐Mini‐2GS switched off) to calibrate the light source (Dreher & Pröhl, 2014; Preißler & Pröhl, 2017). We also measured the irradiance in the field on sunny days at the early afternoon (noon to 2 pm) and late afternoon (4 pm to 6 pm) six times for any given time and locality because in this time period the frogs were most active.

### 2.2 Experiment—coloration plasticity

We conducted a follow‐up experiment with adult yellow‐bellied toads from the large population in Liekwegen in the laboratory of the Institute of Zoology, University of Veterinary Medicine of Hannover, from May 28 to June 8 in 2018. We collected 22 individuals with dip nets from three different but adjacent ponds. We transported the frogs to the institute, each individual in a separate plastic box (370 × 220 × 250 mm) containing natural substrate taken from the ponds and a plastic tube as a hiding spot to reduce animal stress. During the whole study, we kept the frogs separated in these boxes inside a climate chamber under the following conditions: room temperature 20°C, humidity ~60%, illumination with full‐spectrum light (5 am–9 pm), with food *ad libitum* (crickets) provided daily. The weight of the frogs was measured every three days during the experiment.

The habituation phase of the experiment started the day after capturing the frogs. During this phase (day 1–4), we kept all frogs on the natural substrate taken from Liekwegen. Then, in the experimental phase (day 7–11), we randomly assigned the frogs to three treatment groups: “natural substrate” (*n* = 6), “light substrate” (*n* = 8), and “dark substrate” (*n* = 8). The light substrate (“Basissand” by Reptiland) was much lighter and the dark substrate (“Desert sand – black” by EXO TERRA) was slightly darker than the natural substrate. We kept each individual in its plastic box containing the respective substrate on the bottom and a plastic tube, covered with the substrate, for the frogs to hide. About two‐thirds of the area of the box was covered with sand (terrestrial habitat), while the remaining one‐third was covered with sand and water to provide aquatic habitat.

During the whole experiment, including habituation phase (day1–11), we measured the dorsal reflectance of each frog daily, except on days 5 and 6, following the methodology described earlier (“field measurements”). The measurements always started at 14:00 and took approximately two hours in total and approximately 10 min per frog. Since the substrate was changed between 9:00 and 10:00 on day 7, the frogs had spent roughly between 4 and 5 h on the experimental substrate before the measurements. All frogs had to be removed from their boxes during substrate change. Frogs that remained on the natural substrate during the experimental phase were also removed from their boxes to keep the treatment the same for all frogs. Six measurements of the spectral reflectance of each substrate and the irradiance (Figure S1) in the climate chamber were taken and averaged for visual modeling.

### 2.3 Visual modeling

After fieldwork and the experiment, we used the R package “*pavo*” (Maia et al., 2021) to import and process the spectral measurements and to perform visual modeling according to the receptor noise‐limited model developed by Vorobyev and Osorio (1998) (see also Pröhl & Ostrowski, 2011 for details) for the visual system of the starling (Hart, 2001; Hart et al., 1998; Hart & Vorobyev, 2005). Birds constitute one of the few predators of yellow‐bellied toads and various species have been observed to attack (Gollmann & Goldmann, 2012). Birds have a tetrachromatic vision, thus use four types of photoreceptors for color vision: single cones for long, medium, short, and violet or ultraviolet wavelengths (Hart, 2001; Vorobyev & Osorio, 1998). Birds also possess double cones that are thought to impart achromatic vision (Hart & Vorobyev, 2005). First, we calculated the mean spectra of the dorsal coloration for each frog, and for each day, it was measured. We also calculated a mean spectrum for each substrate. The mean spectra were filtered between 300 and 700 nm and resampled by one nanometer. Negative values were converted to zeroes, and spectra were smoothed to remove noise with the *procspec* function (fixneg = “zero,” opt = “smoot,” span = 0.2). The spectral sensitivities for the starling are 362 nm for the ultraviolet‐sensitive receptor (UV), 449 nm for the short wavelength‐sensitive receptor (SWS), 504 nm for the medium wavelength‐sensitive receptor (MWS), and 563 nm for the long wavelength‐sensitive receptor (LWS) (Hart, 2001). The following parameters were incorporated in the *vismodel* and *colddist* function as described in the manual for pavo (Maia et al., 2021): The relative receptor abundances of the starling are 1.0: 3.5: 5.0: 5.5 (Hart et al., 1998), and the default weber fraction for the LWS for chromatic bird vision is 0.1 (Vorobyev et al., 1998) and was used for calculating the color contrast. For calculating the brightness contrast, we used the sensitivities of the double cones (“st.dc”) and the achromatic weber fraction of the starling (*w*_LWS_ = 0.34, Ghim & Hodos, 2006; Olsson et al., 2018).

For the field trial, an average irradiance was calculated for each locality and included in the *vismodel* function for the parameter “illum.” We used the *coldist* function in pavo to calculate the color contrast (Δ*S*) and brightness (Δ*L*) contrast of each frog against its local substrate as well as against the substrate of the other two localities, with values expressed in just noticeable difference (JND) units in the receptor‐noise model. We did this to find out whether the frogs were most cryptic (lowest contrast) on their local substrate and whether differences in contrasts on the different substrates were more pronounced for the chromatic (color) or achromatic channel (brightness contrast) of bird vision.

For the laboratory experiment, we first calculated mean brightness (B2 output from the *summary* function in *pavo*) of each mean spectra per frog and day. This average of reflectance across all 1nm intervals of the spectra is an estimate of how bright the coloration of an animal is, independent from the substrate. Additionally, we calculated the brightness contrast (Δ*L*) of each frog for each day to the substrate it was placed on during the habituation and experimental phases. The obtained visual contrast values are expressed in units JND in the receptor‐noise model. Afterward, we calculated changes in mean and brightness contrast from the habituation phase to the experimental phase.

### 2.4 Statistical analysis

#### 2.4.1 Field measurements

For each substrate derived from the localities Liekwegen, Doberg, and Messingsberg, we compared the dorsal color and brightness contrasts of frogs from the three populations. For that, we fitted linear models to predict the effects of the population (“origin,” categorical with three levels representing the source populations) on the color contrast (Δ*S*) and brightness contrast (Δ*L*) as response variables. Separate tests were conducted for each of the three substrates. We estimated the effect of the frog's source population on each substrate using an ANOVA and estimated the significance of particular comparisons using Tukey post hoc tests.

#### 2.4.2 Experiment on coloration plasticity

The results of the field experiments are suggestive of weak selective pressure on the color of the frog’s dorsum in phenotypic adaptation; hence, in the experiment, we focused on the brightness of individuals. The dorsal mean brightness measurements during the habituation phase were fitted with a linear model and tested with an ANOVA to discard any pre‐existing individual, day, or their interaction effects. To find out whether the experimental substrate treatments had an influence on mean brightness of the frogs, we fitted a linear mixed effects model (estimated using restricted maximum likelihood [REML]) in *lme4* R‐package (Bates et al., 2015) to predict the effects of day (numerical variable), treatment (categorical with three levels representing the light, dark, and natural substrate conditions), and individuals (a random effect term) on the mean dorsal brightness values measured during the experimental treatment (i.e., after excluding the habituation period). We standardized the dataset to obtain standardized parameter estimates (using the *report* R‐package (Makowski et al., 2020) and estimated the effect of the random component by comparing the likelihood of the mixed‐effects model with a similar model without the random component using a likelihood ratio test (LRT). In this and the previous inferences involving linear models, normality, homogeneity of variances, and homoscedasticity assumptions were confirmed using the *check_model* function of the *performance* R‐package (Lüdecke et al., 2021).

The experimental brightness contrasts of each frog (dorsal Δ*L* values against the substrate type it was placed during the experimental phase) were compared against equivalent Δ*L* values measured during the habituation phase. To this effect, Δ*L* values per day were compared with the average Δ*L* values measured during the habituation period using a Dunnett test. The data on individuals of each treatment condition (light or dark substrate) were analyzed separately, and statistically significant differences were assumed when the 95% confidence interval of the difference between means did not include zero. All computations involving R used version 3.6.2.

## 3 RESULTS

### 3.1 Field measurements

The substrates of Doberg, Liekwegen, and Messingsberg differed in coloration (spectral reflectance; Figure 2a). The spectral reflectance of the substrate in Doberg was considerably higher than in Liekwegen und Messingsberg, peaking at 600 nm in the visual spectrum which relates to red coloration (Figure 2a). Likewise, the sampled *B*. *variegata* frogs showed a divergence in dorsal reflectance among populations. Doberg frogs showed a higher spectral reflectance and interindividual variation than frogs from Liekwegen and Messingsberg (Figure 2b).

**FIGURE 3:**
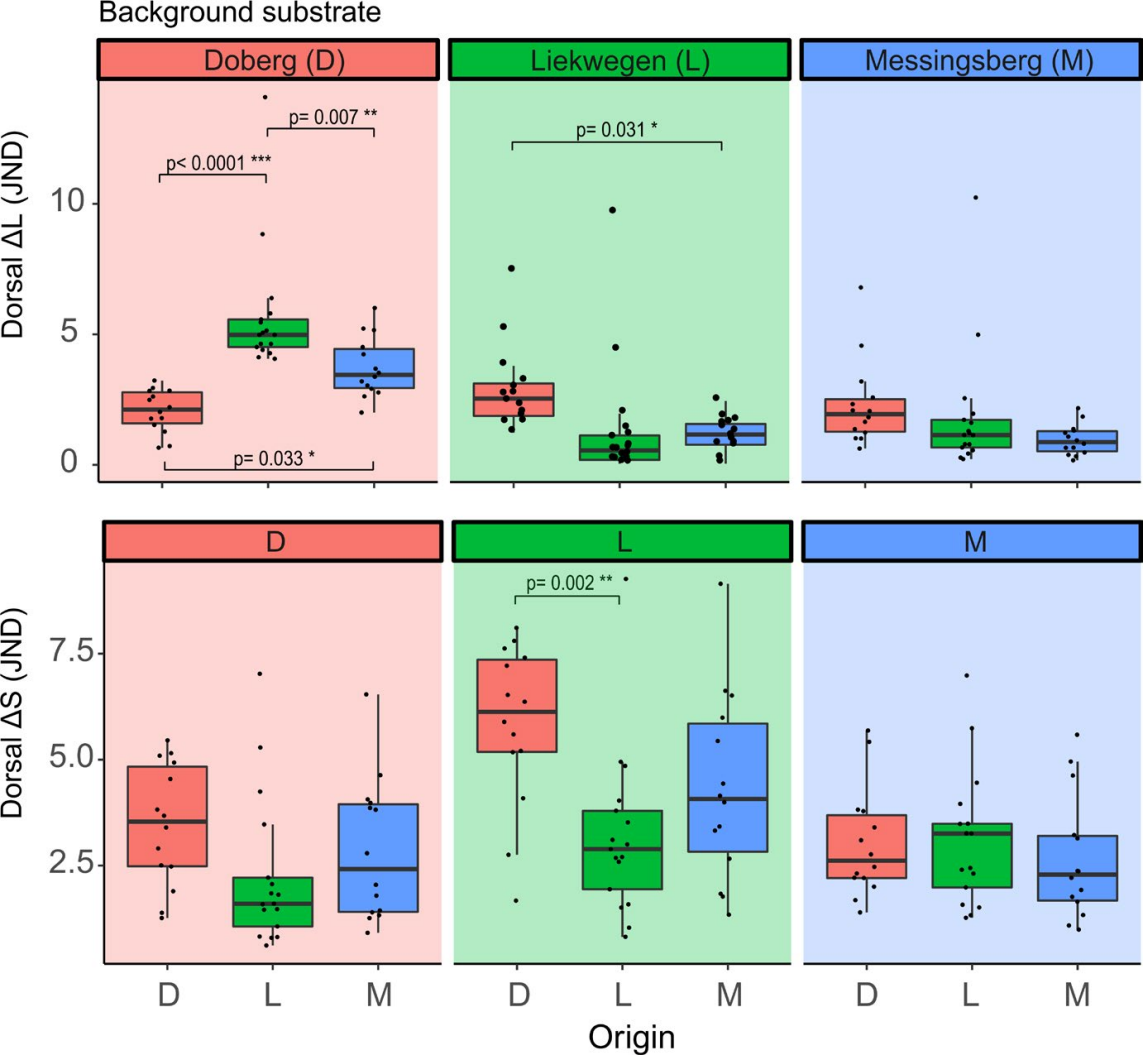
Differences in dorsal brightness (Δ*L*) and color (Δ*S*) contrasts as JND (just noticeable differences) of *Bombina variegata* frogs from the populations of Doberg (D), Liekwegen (L), and Messingsberg (M) calculated using the measurements of the three local substrates. Box color indicates the population origin of the frogs, and the transparent backgrounds indicate the substrate against which the frogs are compared to. Smaller contrast values indicate higher match between individual and substrate. Black dots represent the observed values, boxes span the first and third quartile of the data with the median as a horizontal line, and whiskers span the nonoutlier range

There were statistically significant differences among the mean dorsal brightness contrast (Δ*L*) of the three frog populations against substrates from Doberg (ANOVA: *F*_2,42_ = 17.83, *p* < .0001) and Liekwegen (ANOVA: *F*_2,42_ = 4.22, *p* < .0001) but not Messingsberg (ANOVA: *F*_2,42_ = 2.07, *p* < .138). On Doberg substrate, significant statistical differences were observed among the three comparisons (Tukey post hoc tests *p*‐values < .03), while on Liekwegen substrate, only the comparison between the frogs from Messingsberg and Doberg showed statistical significance (Tukey test *p* = .031) (Figure 3). Overall, frogs exhibited the smallest dorsal brightness contrasts when viewed against their respective local substrate. Frogs from Doberg exhibited the highest brightness contrast to their local substrate, while the frogs from Liekwegen exhibited the best match to its local substrate. Among foreign substrate comparisons, frogs from Liekwegen showed the highest observed brightness contrasts when viewed against Doberg substrate (Figure 3).

The dorsal color contrasts (Δ*S*) showed statistically significant differences among frog populations only when viewed against Liekwegen substrate (ANOVA: *F*_2,42_ = 6.38, *p* < .01) where only the comparison of mean contrasts between Liekwegen and Doberg showed statistical significance (Tukey's test *p* < .05). No statistically significant differences in mean Δ*S* were observed between the three frog populations when viewed against Doberg (ANOVA: *F*_2,42_ = 2.12, *p* = .133) or Messingsberg (ANOVA: *F*_2,40_ = 0.385, *p* = .683) substrates. The highest Δ*S* values were calculated for Doberg frogs against Liekwegen substrate and the lowest values for frogs from Liekwegen against Doberg substrate (Figure 3).

### 3.2 Experiment—coloration plasticity

**FIGURE 4:**
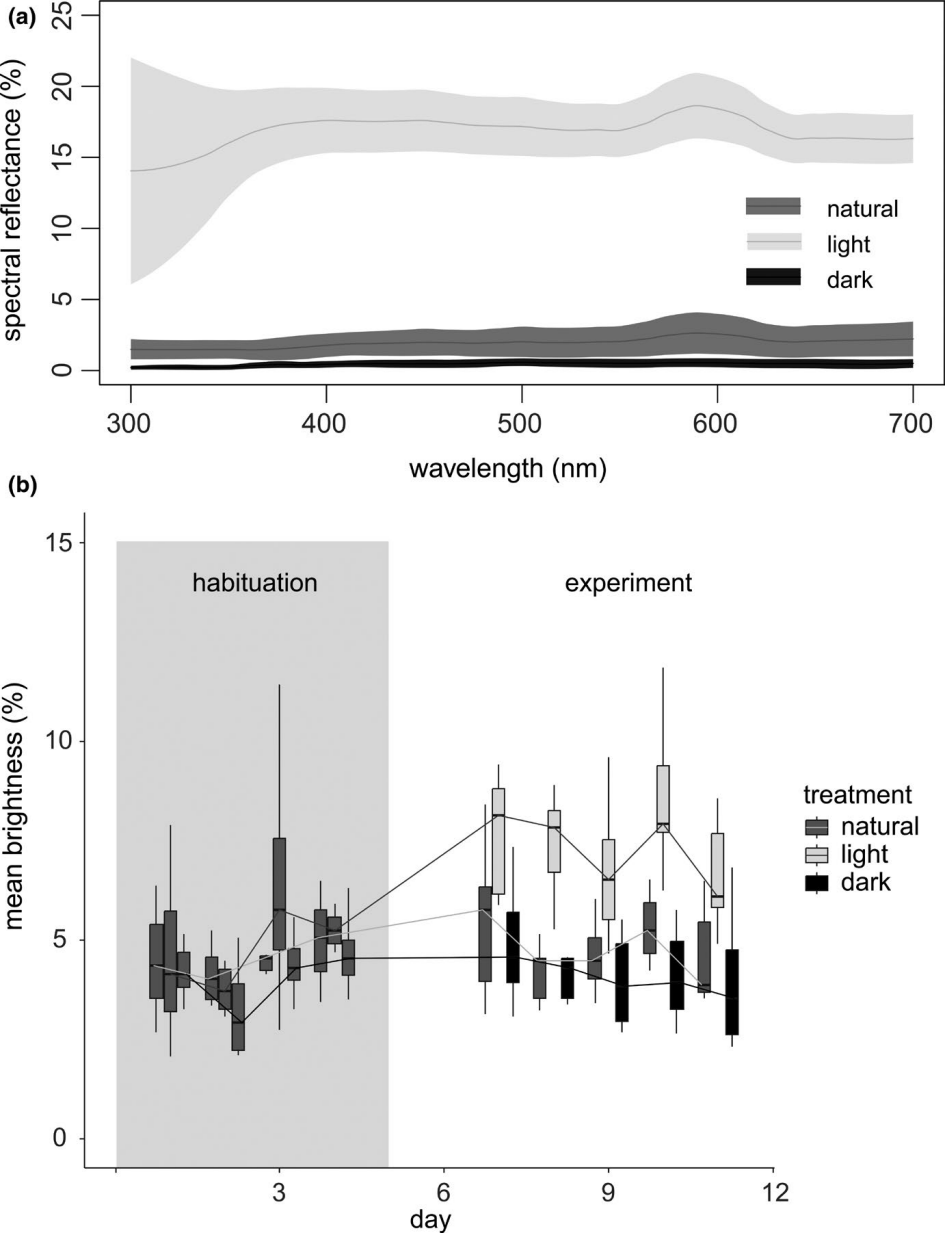
(a) Spectral reflectance for the visible range of wavelengths (nm) of the natural Liekwegen substrate (natural) and two artificial substrates (light, dark) used in the experiment. (b) Boxplots of mean brightness values measured on *Bombina variegata* frogs’ dorsa while kept on terraria with the three substrate types during habituation and experimental treatments

The spectral reflectance curves for the light substrate were much higher across the whole visual spectrum than the curves for the other two substrates. The reflectance curve for the dark substrate was only marginally lower than the curve for the natural substrate from Liekwegen (see spectral reflectance in Figure 4a).

#### 3.2.1 Mean brightness

In the habituation phase, when all individuals were on natural substrate, the mean brightness of the frog's dorsal skin varied (mean: 4.6, minimum–maximum: 1.8–11.4) but showed no statistically significant differences between days (*F*_3,84_ = 3.08, *p* = .08), individuals (*F*_3,84_ = 1.27, *p* = .26), or their interaction (*F*_3,84_ = 0.07, *p* = .78) (Figure 4b). The linear mixed model applied to the data from the experimental phase, that is, excluding the habituation period, had a substantial explanatory power (conditional *R* ^2^ = 0.53) most of it related to the (fixed) effects of treatment and day (marginal *R* ^2^ = 0.45). Under this model, we detected a positive and highly significant effect of the experimental light substrate treatment on the mean brightness of the frogs (*β* = 3.06, SE = 0.57, 95% CI [1.95, 4.17], *β*_std_ = 1.24, *p* < .001). The effect of the dark substrate treatment on the mean brightness was negative but not significant (*β* = −0.64, SE = 0.57, 95% CI [−1.75, 0.47], *β*_std_ = −0.26, *p* = .256). The number of days since the start had a very small and not significant effect on the total brightness of frogs on all substrates (*β* = −0.10, SE = 0.12, 95% CI [−0.32, 0.13], *β*_std_ = −0.06, *p* = .393) (Figure 4b). The effect of individual was negligible as models with and without this random effect term performed comparably (LRT: *Χ* ^2^ = 2.38, *p* = .122).

#### 3.2.2 Brightness contrasts

**FIGURE 5:**
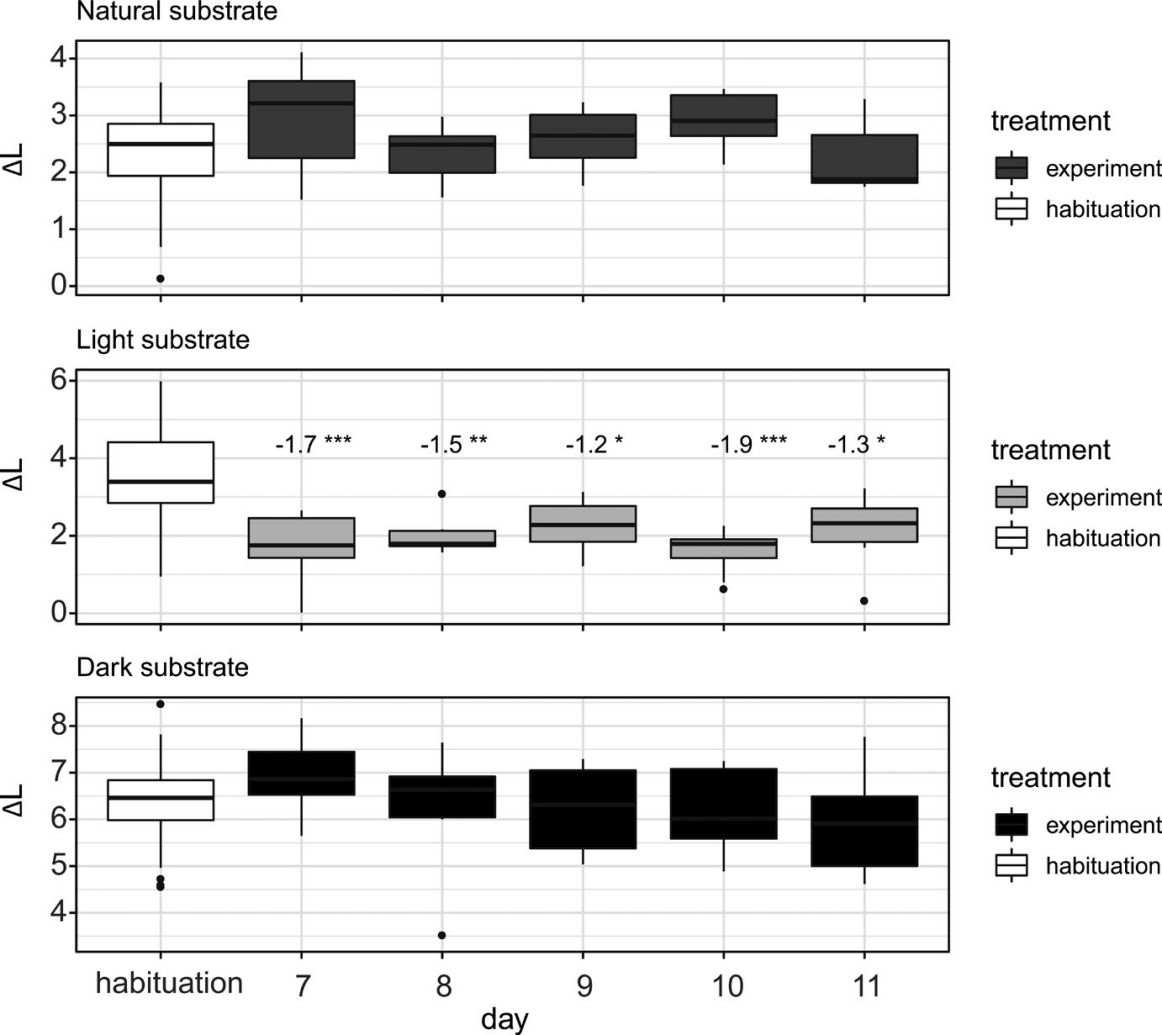
Variation in brightness contrast (Δ*L* as JND (just noticeable differences), shown as boxplots) of *Bombina variegata* individuals to each substrate type during each day of the experimental phase (gray‐black) and the reference values calculated for that same substrate type in the habituation phase (white). These brightness contrasts are thus indicative of the extent of the induced coloration change (adaptation) of the frogs and were calculated between each experimental substrate and frog dorsum reflectance spectra using an avian (predator) visual model. Significant differences were observed only in the light substrate treatment, and values on top of boxes indicate the effect sizes estimated by the Dunnett test (* *p* < .05; ** *p* < .01; *** *p* < .001)

We observed changes in the brightness contrasts of the skin of the frogs between the habituation and the experimental phase for the light substrate treatment only (Figure 5; Table 1). The brightness contrast (i.e., conspicuity) to the light substrate decreased significantly, and the change was already observable from the first day of the treatment and was stable throughout all the five experimental days. The frogs placed on dark and natural substrates showed a very small and nonsignificant change in total brightness contrast (Figure 5; Table 1).

**TABLE 1:** Results of the Dunnet test comparing the means of brightness contrast of *Bombina variegata* individuals to each substrate type during the experimental phase against the respective reference values measured during the habituation phase

Substrate	Day	Estimate	Low 95%CI	Upper 95%CI
Natural	7	0.61	−0.32	1.54
	8	−0.01	−0.94	0.92
	9	0.24	−0.68	1.17
	10	0.57	−0.36	1.49
	11	−0.10	−1.03	0.83
Light	7	**−1.71**	**−2.74**	**−0.68**
	8	**−1.49**	**−2.52**	**−0.46**
	9	**−1.20**	**−2.23**	**−0.17**
	10	**−1.88**	**−2.90**	**−0.85**
	11	**−1.32**	**−2.35**	**−0.30**
Dark	7	0.52	−0.51	1.55
	8	−0.09	−1.12	0.94
	9	−0.17	−1.20	0.85
	10	−0.24	−1.27	0.79
	11	−0.41	−1.44	0.62

Note

Significant results (95% confidence intervals [CI] excluding zero) are highlighted in bold.

#### 3.2.3 Size and weight

The mean size of the frogs was 3.7 cm (SD = 0.46) and the mean weight 20.3 g (SD = 7.08). The frogs lost a bit of weight from the first day of habituation (mean = 21.2, SD = 7.84) to the last day (19.9, SD = 7.12) of the experiment (paired *t*‐test: *t* = 4.44, *p* = .0002, mean difference = 1.22 g).

## 4 DISCUSSION

Phenotypic adaptation is a well‐known evolutionary process to enhance survival in prey species, such as amphibians (Michimae, 2006; Wente & Phillips, 2003), reptiles (McLean et al., 2014), mice (Hoekstra et al., 2005), fish (Stevens et al., 2014), and invertebrates (Cuthill et al., 2005). The present study demonstrates that the cryptic dorsal coloration of the yellow‐bellied toad conceals the frogs in their natural environment from avian vision. This concealment is achieved by a small color and brightness contrast between the dorsal skin of the frogs and their natural substrates. However, the magnitude of phenotypic adaptation in coloration was not equal among sampled populations. While the brightness contrast between the frog's skin and the substrate was low in Liekwegen and Messingsberg, the Doberg population showed a moderate brightness contrast even to its own substrate, indicating that crypsis is not maximized. The substrate in Doberg is relatively light compared to many other localities where yellow‐bellied toads occur (H. Pröhl, personal observation), and even though the Doberg habitat is suitable to reproduce and survive, the species might physiologically not be able to increase the reflection of light from the skin to appear completely cryptic. In contrast to frogs from other locations, the Doberg frogs were repeatedly observed to cover themselves with pond soil. This could hint at a perception of the imperfect match of their dorsal coloration to the substrate. Amphibians are known to employ mechanisms linked to light‐controlled skin pigmentation. For instance, melanopsin located in skin pigment cells can directly induce skin darkening to enhance thermoregulation and ultraviolet protection. Furthermore, melanopsin‐expressing eye cells control neuroendocrine circuits to mediate background adaptation in response to surface‐reflected light (Bertolesi & McFarlane, 2018).

Multiple factors affect the coloration of animals. Natural and sexual selection in which predators and mates respectively select for a certain phenotype (Dreher & Pröhl, 2014; Duarte et al., 2017; Gade et al., 2016) can influence body coloration over longer evolutionary times. However, physiological mechanisms can alter the coloration (Nilsson Sköld et al., 2013; Rudh & Qvarnström, 2013) quickly and temporarily. In our second experiment, we demonstrated that rapid physiological adjustments in dorsal coloration play a role in phenotypic adaptation in *B*. *variegata*. Regulated by an interplay of genetic, environmental, and endocrine factors, animals change color by moving pigment‐containing melanosomes within cells during reproduction or rapid adaptation to changing environments (Aspengren et al., 2009; Duarte et al., 2017; Nilsson Sköld et al., 2013). We observed a difference in the time frogs needed to adjust to lighter and darker substrates. Frogs transferred to a lighter substrate adjusted within one day to a higher brightness which on average did not change over the trial. The higher brightness of the skin resulted in a significant decrease of the brightness contrast to the light substrate, that is, by this, the frogs on light substrate were able to enhance their crypsis to avian predators quickly. The frogs transferred to a darker substrate steadily decreased in brightness until the end of the experiment and were not able to decrease the brightness contrast to the substrate significantly. However, the differences in spectral reflectance for the natural and dark substrate were possibly too minor for the frogs to trigger a change in brightness. The ability to change coloration to match lower brightness values should be studied more thoroughly in future studies. However, different functions of organelles and pigments within melanophores might explain the difference in time the skin needs to adapt to lighter versus darker substrates. Melanophores are able to rapidly aggregate and disperse melanosomes (Fujii, 2000; Ligon & McCartney, 2016). The skin gets lighter when melanosomes contract and concentrate within the melanophores. In addition to dispersion of melanosomes, it might be necessary to produce more melanin, which depends on the speed of melanin gene expression in cell organelles during adaptation to darker substrates (Leclercq et al., 2009). The longer the adjustment takes, the higher the predation risk for frogs on darker substrates. This important conservation issue needs consideration when translocating individuals.

The yellow‐bellied toad is endangered in Germany (Kühnel et al., 2009) and critically endangered in the German state of Lower Saxony (Podloucky & Fischer, 1994). During reproduction, the frog relies on small ephemeral puddles located in open areas lacking vegetation, that is, quarries or abandoned military areas (Kwet, 2015). Habitats suitable for reproduction have been lost in the landscape mainly due to expanding agriculture, leading to isolated and small populations with low genetic diversity. The ability to adapt to a certain environment is crucial for the survival and maintenance of a population. Organisms are better enabled to react to environmental stress when large in population size and high in genetic variation (Agashe et al., 2011; Bell & Gonzalez, 2009; Pujol & Pannell, 2008; Willi & Hoffmann, 2009). It was shown that inbred populations have slower responses to environmental pressures especially in new habitats (Reed et al., 2003; Wade et al., 1996). The low capacity of Doberg frogs in phenotypic adaptation (Figure 3) could be correlated to the small population size and low genetic diversity (*H*_o_ = 0.30, *H*_e_ =0.41), while the population in Liekwegen is larger and more divers (*H*_o_ = 0.49, *H*_e_ = 0.51) and seems to be better adapted to the local substrate (Pröhl et al., 2021). In the experiment however, Liekwegen frogs displayed comparably high brightness contrasts to even their natural substrate (Figure 5), suggesting that yellow‐bellied toads in general are physiologically limited in changing color.

In recent conservation approaches, individuals or subpopulations of the fire‐bellied toad (*Bombina bombina* *)* were relocated to support or mirror populations suffering from genetic erosion (Schröder et al., 2012). Relocation to new habitats could decrease the efficacy of dorsal camouflage of individuals that are adapted to different substrates and consequently increase predation. We found that the frogs from one population were able to adapt their dorsal coloration to a lighter substrate; however, it is still unclear how frogs from other populations would react to habitats with either darker or lighter substrates. The magnitude of coloration plasticity might vary among populations within the same species. Ideally, in conservation programs, the capacity of dorsal color change is measured before releasing frogs into a new habitat. For example, in a group of shore skinks, strong selection for camouflage seems to have produced a high match in color pattern between the animals and the new habitat after one year of the translocation, however at the cost of low survivorship of mismatched individuals (Baling et al., 2016).

## 5 CONCLUSION

We find strong evidence for coloration plasticity in *B*. *variegata*, significantly pronounced in dorsal brightness. However, we also observe that this ability might be limited in certain populations. Future studies should include additional populations to explore the physiological limit on color change as well as genetic diversity estimates to infer the influence of genetic diversity on the potential of rapid phenotypic adaptation. These matters have not been studied but have important implications for the conservation of this and other endangered species.

## CONFLICT OF INTEREST

The authors declare that no conflict of interest exists.

## AUTHOR CONTRIBUTIONS

**Kathleen Preißler:** Formal analysis (equal); Visualization (equal); Writing‐original draft (equal). **Ariel Rodríguez:** Conceptualization (equal); Formal analysis (equal); Supervision (equal); Visualization (equal); Writing‐original draft (equal). **Heike Pröhl:** Conceptualization (equal); Methodology (lead); Project administration (lead); Supervision (equal); Writing‐original draft (equal).

## Supporting information

Supplementary Material

## Data Availability

Original spectrometer measurements, associated metadata, and the R scripts used for processing, statistical analyses, and plotting are available on: https://github.com/ArielRodrig/Bombina_variegata; https://doi.org/10.5281/zenodo.5647952.
