# Supplementary material for: Evidence for coloration plasticity in the yellow‐bellied toad, Bombina variegata
Source: Ecol Evol. 2021 Nov 30;11(23):17557–67. doi: 10.1002/ece3.8391 (PMC8668782; doi:10.1002/ece3.8391)
Supplement: Supplementary file 1 — Supplementary Material [file ECE3-11-17557-s001.pdf]

Supplementary Materials for:

Evidence for coloration plasticity in the yellow-bellied toad, *Bombina variegata*

Kathleen Preißler, Ariel Rodríguez, Heike Pröhl

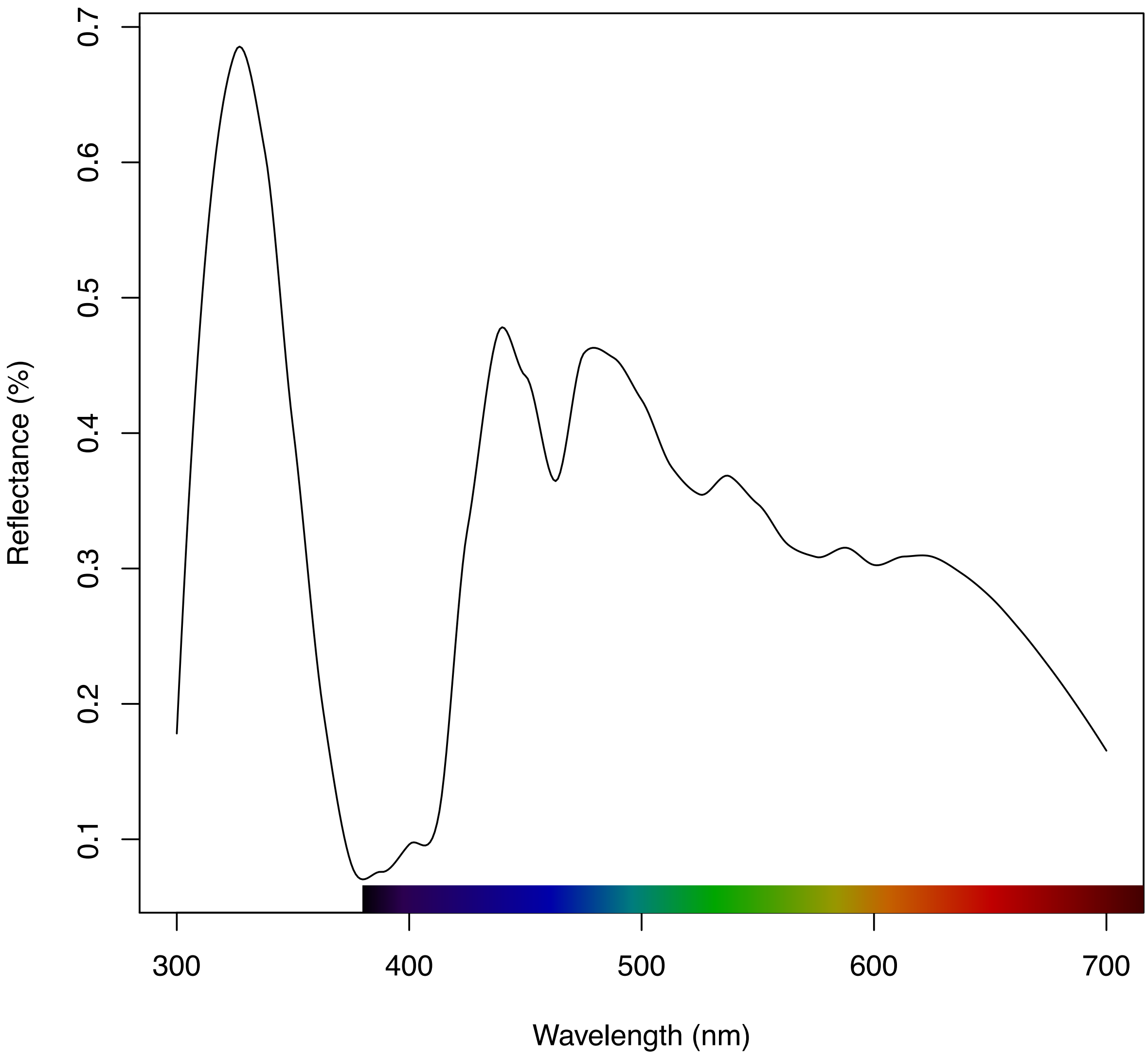

Figure S1: Irradiance (spectral reflectance in %) for the visible range of wavelengths (nm) in the climate chamber.
